# Supplementary material for: Differential Immunomodulatory Effects of Head and Neck Cancer-Derived Exosomes on B Cells in the Presence of ATP
Source: Int J Mol Sci. 2022 Nov 21;23(22):14446. doi: 10.3390/ijms232214446 (PMC9693630; doi:10.3390/ijms232214446)
Supplement: Supplementary file 1 [file ijms-23-14446-s001.zip › ijms-1956469-supplementary.pdf]

Supplementary Figures

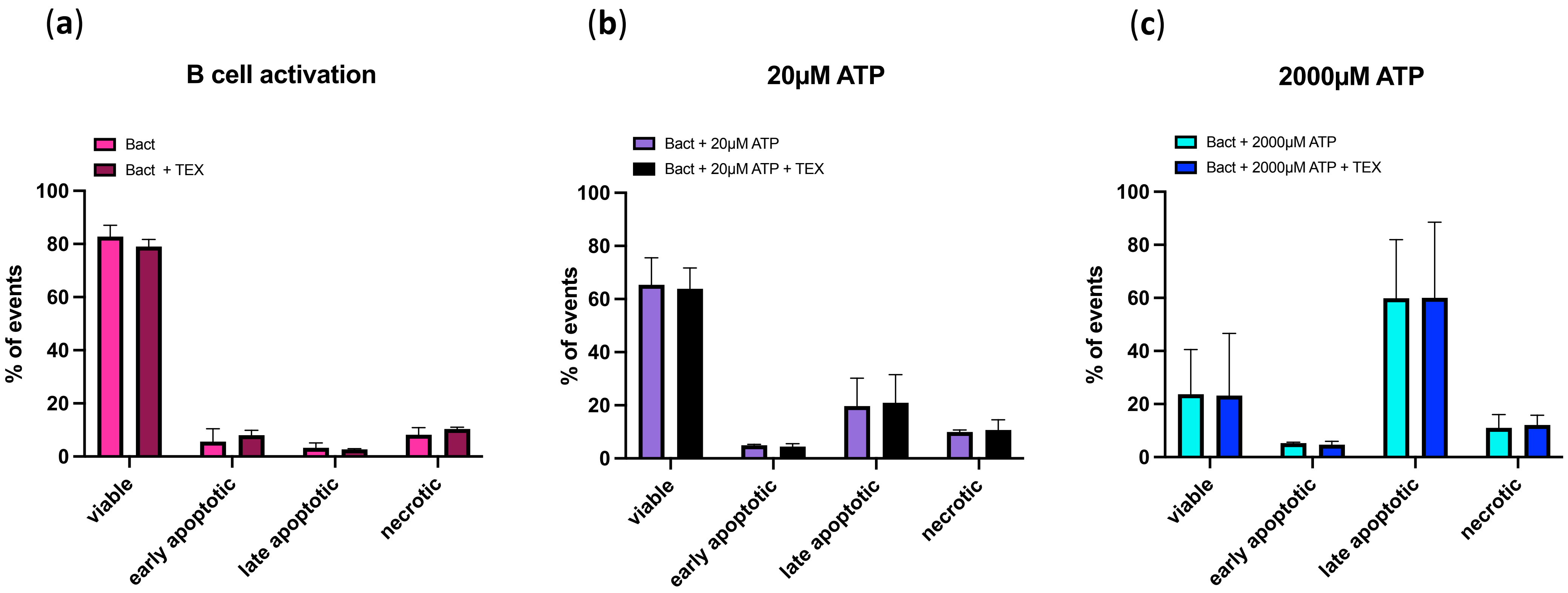

Figure S1. Bar charts showing the effect of TEX on cell death in activated B cells (Bact) with or without ATP based on flow cytometry data of Annexin V and Propidium Iodide (PI) staining as seen in figure 5a. **(a)** Bar charts showing no significant changes by TEX on the rates of viable or dead cells in Bact. **(b)** Bar charts showing no significant changes by TEX on the rates of viable or dead cells in Bact co-incubated with 20μM ATP. **(c)** Bar charts showing no significant changes by TEX on the rates of viable or dead cells in Bact co-incubated with 2000μM ATP. Statistical significance was determined using a 2-way ANOVA Tukey's multiple comparisons test. The mean of four independent experiments with standard deviation are presented.

(a)

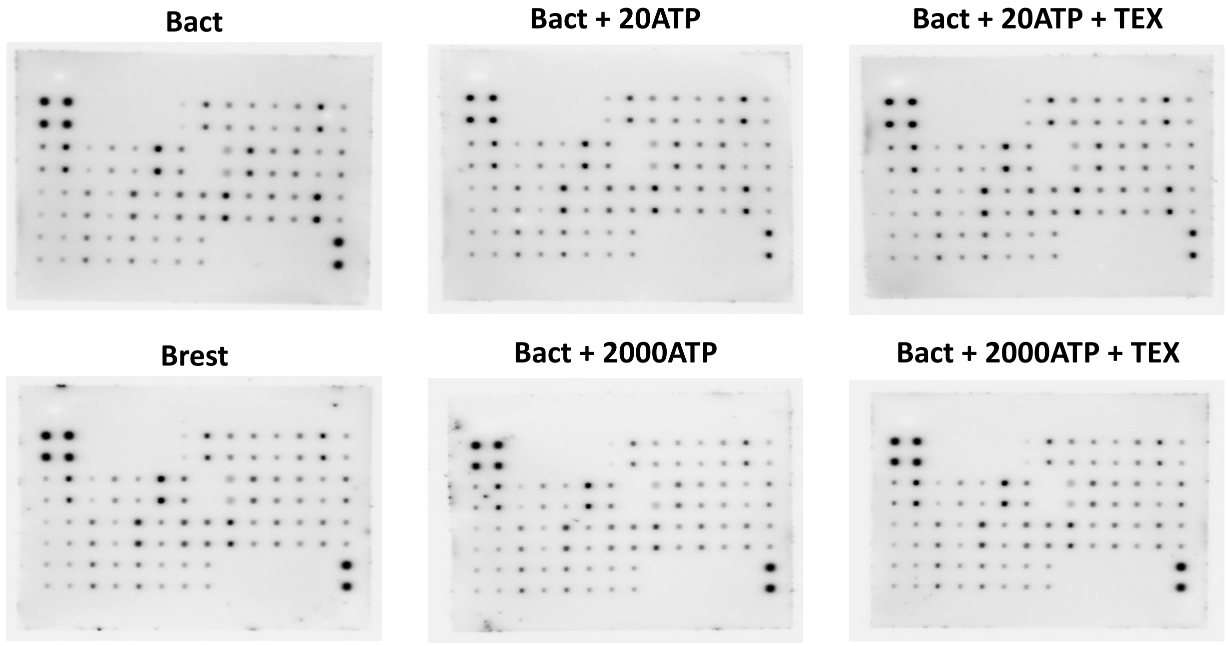

(b)

Pos	Pos	Neg	Neg	BLANK	BLANK	Bad	Bax	Bcl-2	Bcl-w	BID	BIM	Caspase 3	Caspase 8
Pos	Pos	Neg	Neg	BLANK	BLANK	Bad	Bax	Bcl-2	Bcl-w	BID	BIM	Caspase 3	Caspase 8
CD40	CD40L	cIAP-2	cytoC	DR6	Fas	FasL	BLANK	HSP27	HSP60	HSP70	HTRA	IGF-I	IGF-II
CD40	CD40L	cIAP-2	cytoC	DR6	Fas	FasL	BLANK	HSP27	HSP60	HSP70	HTRA	IGF-I	IGF-II
IGFBP-1	IGFBP-2	IGFBP-3	IGFBP-4	IGFBP-5	IGFBP-6	IGF-1sR	Livin	p21	p27	p53	SMAC	Survivin	sTNF-R1
IGFBP-1	IGFBP-2	IGFBP-3	IGFBP-4	IGFBP-5	IGFBP-6	IGF-1sR	Livin	p21	p27	p53	SMAC	Survivin	sTNF-R1
sTNF-R2	TNF-α	TNF-β	TRAILR-1	TRAILR-2	TRAILR-3	TRAILR-4	XIAP	BLANK	BLANK	Neg	Neg	Neg	Pos
sTNF-R2	TNF-α	TNF-β	TRAILR-1	TRAILR-2	TRAILR-3	TRAILR-4	XIAP	BLANK	BLANK	Neg	Neg	Neg	Pos

Figure S2. Dot blots of 43 apoptosis-associated proteins and controls. **(a)** Representative apoptosis dot blot results of the experiments with isolated B cells in six different conditions (Brest: resting B cells; Bact: activated B cells; Bact with 20µM or 2000µM ATP, and Bact with 20µM or 2000µM ATP and TEX). **(b)** Array map for the 43 apoptosis markers and controls.

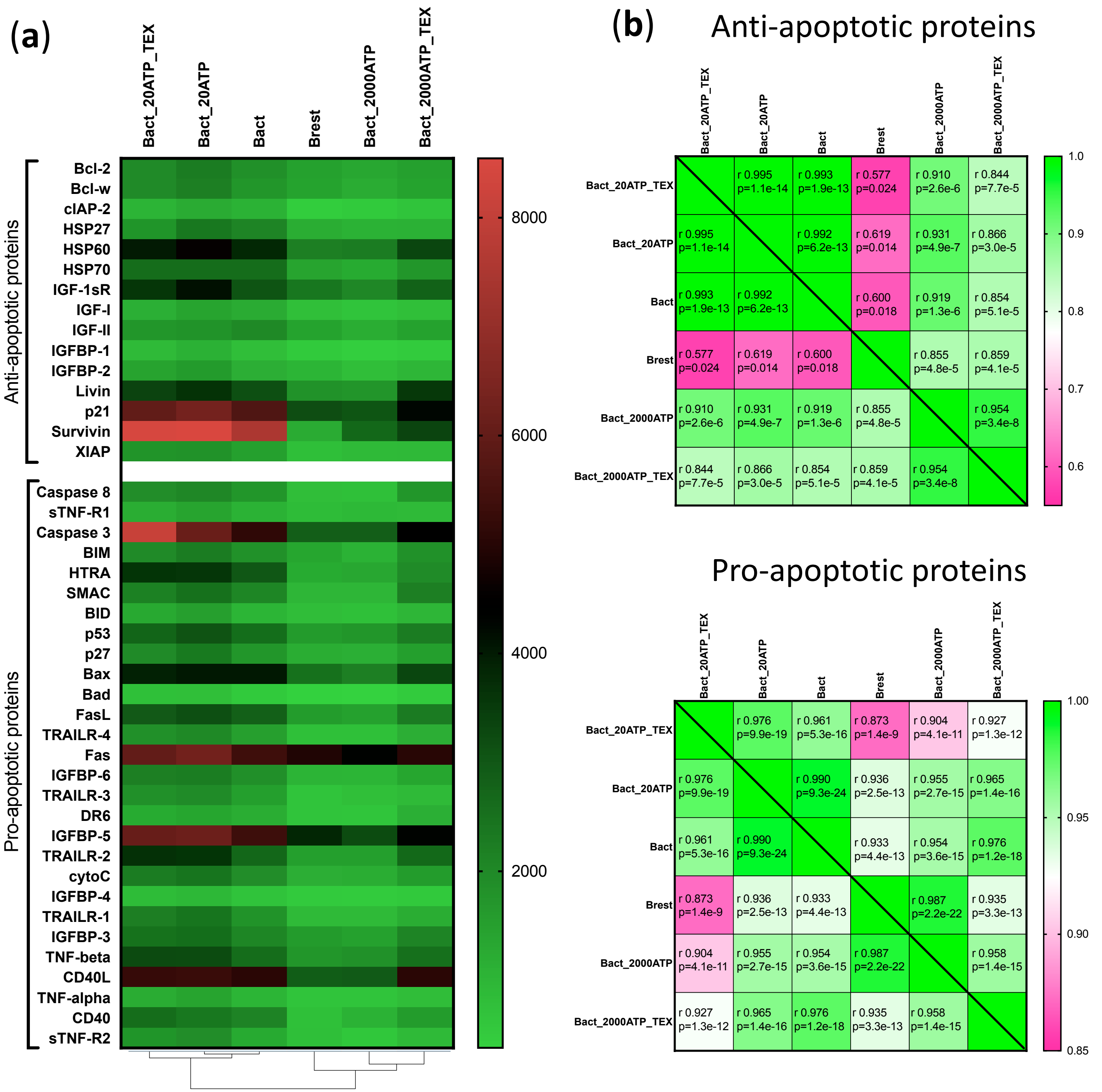

Figure S3. Heatmaps showing the expression of the 43 apoptosis-associated proteins separated as pro- and anti-apoptotic proteins as a mean of four independent experiments in isolated B cells in six different conditions (Brest: resting B cells; Bact: activated B cells; Bact with 20µM or 2000µM ATP, and Bact with 20µM or 2000µM ATP and TEX). **(a)** Semi-supervised clustering uncovered 2 groups: Bact and Bact with 20µM ATP with or without TEX versus Brest and Bact with 2000µM ATP with or without TEX. Distance measuring method: Pearson. Clustering method: Average Linkage. **(b)** Heatmap showing the protein expression as an overall mean. Differences between each group were highly significant. For the anti-apoptotic proteins, the biggest differences were seen between Brest and the Bact or Bact + 20ATP +/- TEX. For the pro-apoptotic proteins, these differences were less pronounced. Instead, the differences were greater between Bact + 20ATP + TEX and Brest or Bact + 2000ATP +/- TEX. The matrix of the anti-apoptotic proteins showed lower r-values than the one of the pro-apoptotic (see legend bar). Pearson correlation matrix with their respective r-values and statistical significances (p-values).

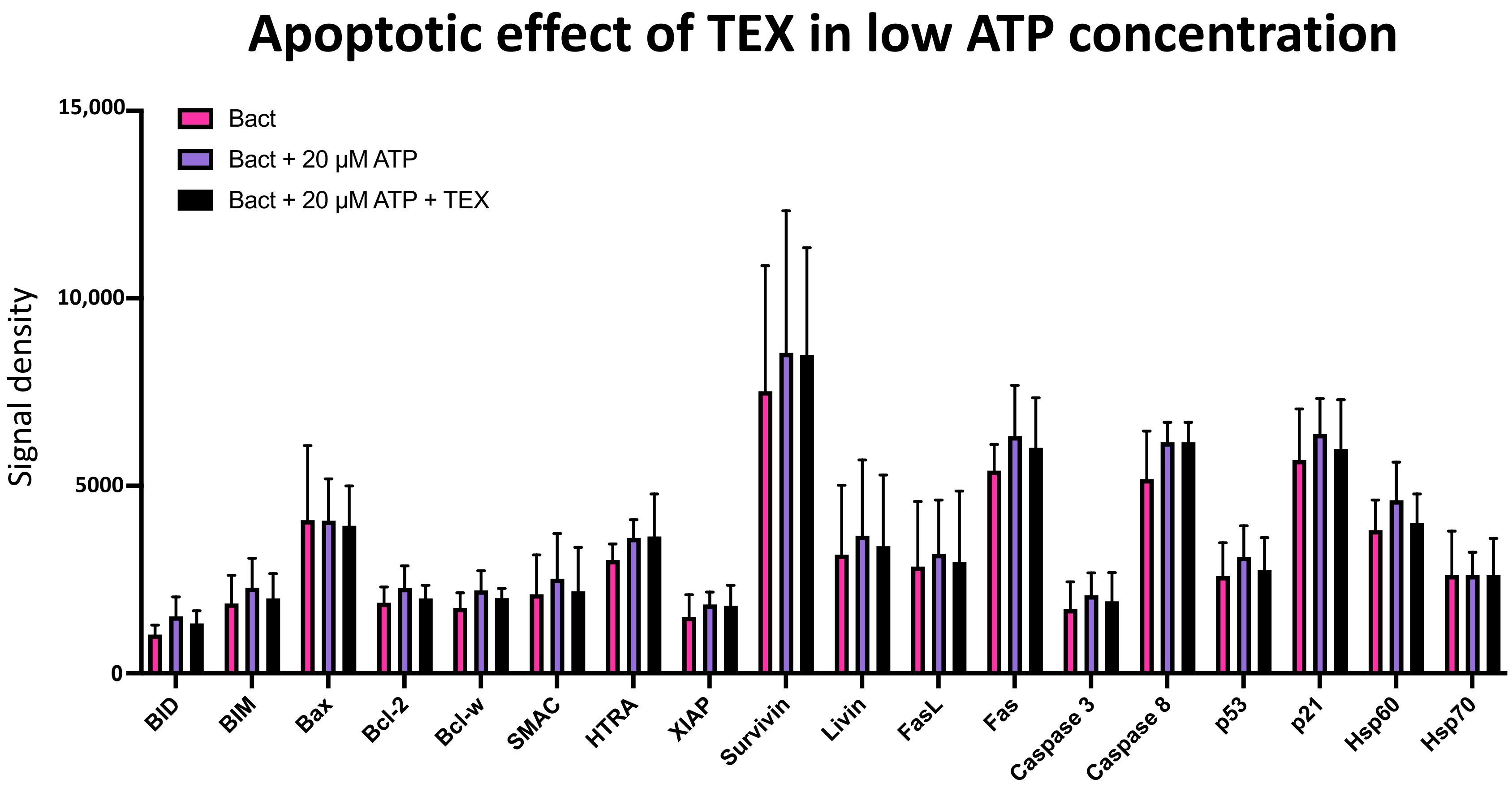

Figure S4. Bar charts comparing the expression of 18 of the pro- and anti-apoptotic proteins shown in figure 6b in activated B cells (Bact), Bact + 20μM ATP and Bact + 20μM ATP + TEX. No significant changes detected. Significance determined by a 2-way ANOVA Tukey's multiple comparisons test.

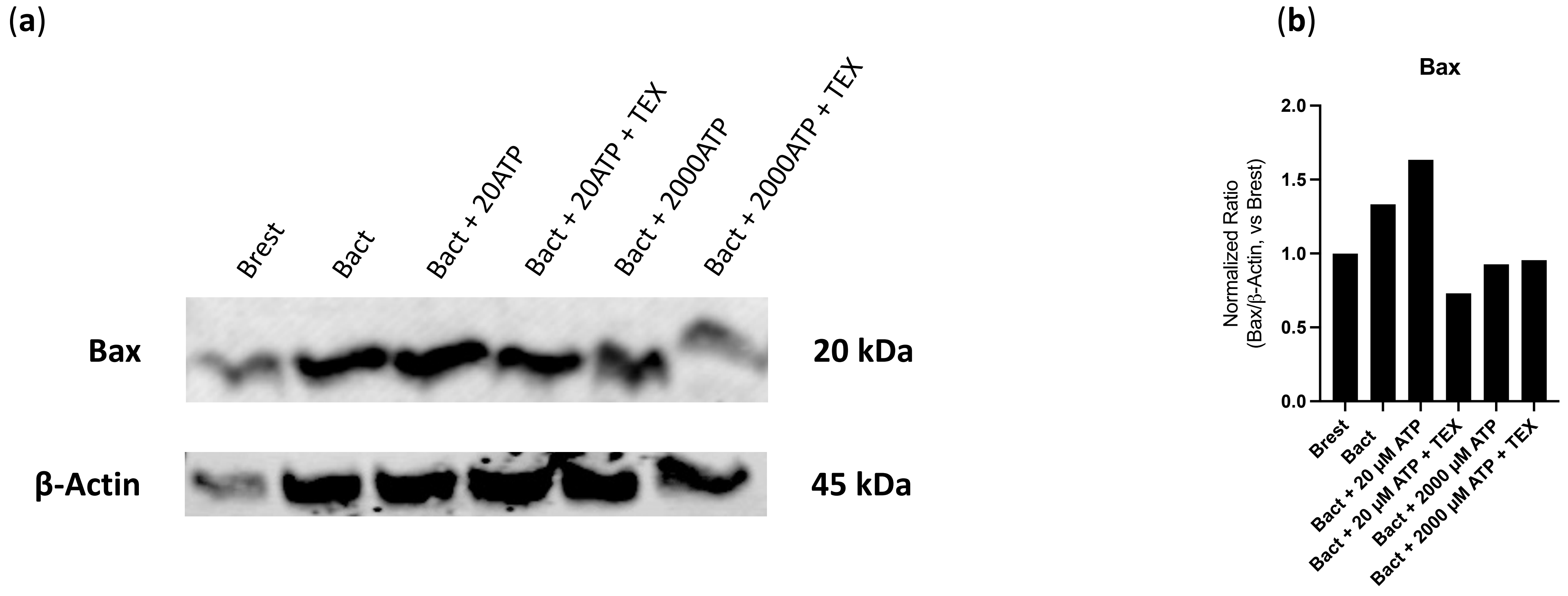

Figure S5. **(a)** Western Blot analysis of the pro-apoptotic protein Bax in resting B cells (Brest) or activated B cells (Bact), including different culture interventions. **(b)** Quantification of western blot analysis of Bax in Brest or Bact, including different culture interventions. Activation of B cells increased Bax expression. Low amounts of ATP (20µM ATP) further increased Bax expression. However, high amounts of ATP (2000µM ATP) strongly decreased Bax expression. Whereas TEX addition to low amounts of ATP starkly decreased Bax expression, TEX addition to high amounts of ATP maintained expression levels with a possible minor increase.

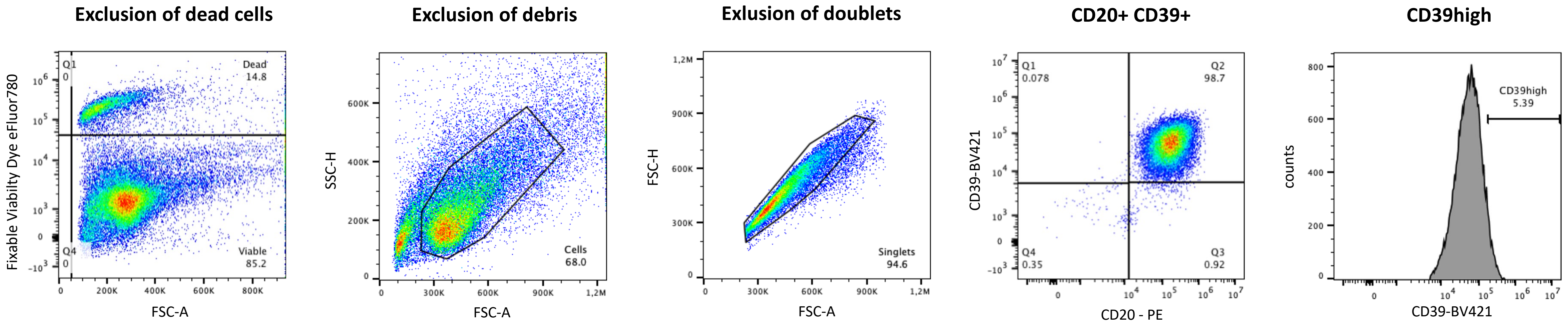

Figure S6. Flow cytometry gating strategy for CD39+ B cells and CD39high B cell subset in anti-CD19 magnetically separated B cells. CD39high B cells were defined as the 5% of CD39+ B cells with the highest fluorescence intensity for CD39 following B cell activation [22].
